# Evaluation of the Antibacterial Activity of Cinnamic Acid and Its Derivatives: Synergistic Effects with Cloxacillin

**DOI:** 10.3390/molecules30030660

**Published:** 2025-02-02

**Authors:** Tomasz Zawiła, Denis Swolana, Jakub Rok, Zuzanna Rzepka, Robert D. Wojtyczka

**Affiliations:** 1 Department of Microbiology, Faculty of Pharmaceutical Sciences in Sosnowiec, Medical University of Silesia, ul. Jagiellońska 4, 41-200 Sosnowiec, Poland; d200969@365.sum.edu.pl (T.Z.); dswolana@sum.edu.pl (D.S.); 2 Department of Pharmaceutical Chemistry, Faculty of Pharmaceutical Sciences in Sosnowiec, Medical University of Silesia, ul. Jagiellońska 4, 41-200 Sosnowiec, Poland; jrok@sum.edu.pl (J.R.); zrzepka@sum.edu.pl (Z.R.)

**Keywords:** antimicrobial activity, biofilm, cinnamic acid, *Staphylococcus epidermidis*, confocal microscopy

## Abstract

*Staphylococcus epidermidis* is a widely prevalent microorganism whose key virulence factors include a high ability to adhere to synthetic surfaces and the capacity to form biofilms. The widespread distribution of multidrug-resistant strains (e.g., MRSE—methycillin-resistant *Staphylococcus epidermidis*) compels researchers to explore new therapeutic approaches. Cinnamic acid and its derivatives are common plant-derived compounds known for their broad range of biological activities, including antimicrobial properties. The phenotypic assays conducted in this study revealed a strong anti-biofilm activity of the investigated compounds. Confocal laser scanning microscopy allowed for the visualization of structural changes within the biofilm and facilitated the assessment of bacterial cell viability in relation to the concentration of the tested substances.

## 1. Introduction

*Staphylococcus epidermidis* (*S. epidermidis*) is a significant microorganism that constitutes part of the human skin’s microbiota. It plays a crucial role in maintaining homeostasis, preventing the adhesion of potential pathogens, and positively influencing regenerative processes [1]. However, in recent years, this microorganism has been recognized as one of the most important pathogens associated with hospital-acquired infections. These infections are not limited to intensive care units but also occur in other facilities such as outpatient clinics, nursing homes, and similar settings. Key virulence factors include a high percentage of antibiotic-resistant strains, the ability to adhere to biomaterials, and biofilm production [2,3]. A biofilm is a structure composed of bacteria suspended in an extracellular matrix and attached to either biotic (e.g., tissue) or abiotic surfaces (e.g., medical devices). The majority of the biofilm mass (85-90%) consists of proteins, sugars, and nucleic acids that form the matrix, while the remaining mass (10-15%) comprises bacterial cells suspended in a three-dimensional “universe”. The existence of bacteria within the biofilm structure significantly increases their resistance to adverse external factors, such as antibiotics or the host’s immune system [4,5]. The ability to form biofilms is a feature observed in various microorganisms and depends on a range of biological, chemical, and physical factors, with the contribution of each factor varying based on environmental conditions.

Plastics used in medical care are susceptible to the adhesion of macromolecules such as albumins, glycoproteins, and immunoglobulins. Surfaces coated with these molecules provide an environment conducive to microbial colonization [6]. Various molecules are responsible for microbial adhesion, and among *Staphylococcus* spp., the most significant appears to be polysaccharide intercellular adhesin (PIA). The synthesis of this compound is controlled by the *icaADBC* operon [5].

The largest and most frequently used group of antibiotics is β-lactams. Antibiotics in this group have been known for nearly 100 years (discovered by Fleming in 1928). These agents are characterized by a broad spectrum of activity and an excellent safety profile. Unfortunately, the widespread use of these antibiotics, coupled with irresponsible antibiotic policies, has led to a rapid increase in the number of multidrug-resistant strains. In the case of *Staphylococci*, the primary resistance mechanism involves the alteration of the antibiotic’s target site [7].

A high proportion of methicillin-resistant *Staphylococci* (strains with alterations in PBP proteins), the ability to produce biofilms and the poor overall health of patients make hospital-acquired infections challenging to treat and a serious threat to health and life. This situation necessitates the search for new therapeutic strategies and the combination of existing drugs with natural substances and their derivatives to develop new and effective treatments.

**Figure 1:**
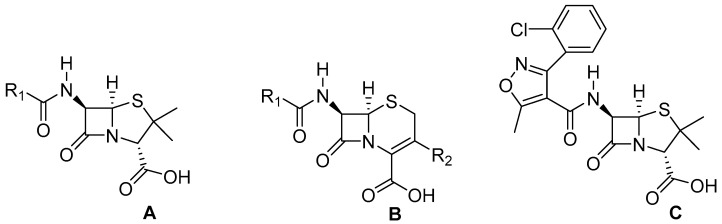
General structural formulae of: (**A**) Penicillins; (**B**) Cephalosporins; (**C**) Cloxacillin; R^X^—Substituent.

**Figure 2:**
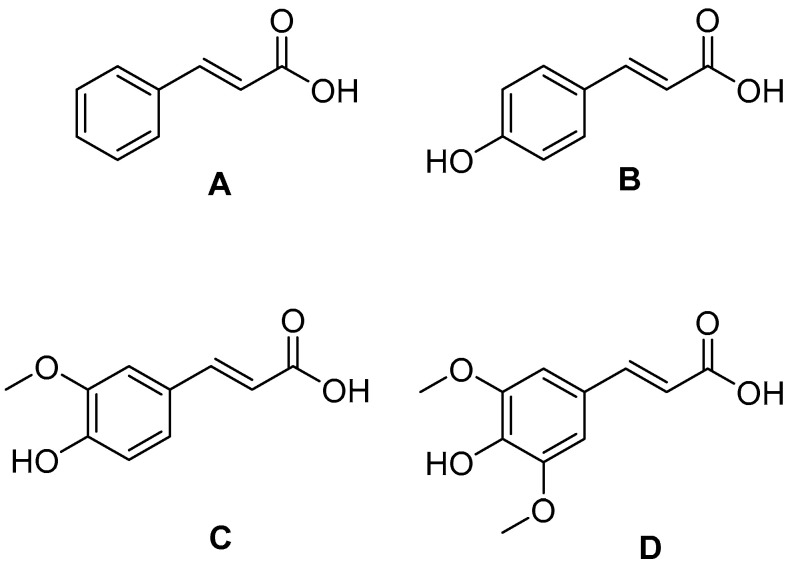
Structural formulae of selected acids: (**A**) cinnamic acid; (**B**) p-coumaric acid; (**C**) ferulic acid; (**D**) sinapic acid.

One group of compounds with broad-spectrum antimicrobial activity includes cinnamic acid and its derivative acids. Cinnamic acid and its derivatives are carboxylic acids widely distributed in the plant kingdom. These compounds play an essential role in plant metabolism and serve as substrates in the biosynthesis of compounds such as anthocyanins, coumarins, flavonoids, and tannins [8]. They exhibit various biological activities, including anti-inflammatory, antioxidant, neuroprotective, and antimicrobial effects. These substances are active against Gram-positive and Gram-negative bacteria, yeasts, molds, and mycobacteria, and they also exhibit antiparasitic properties [8,9,10,11]. These compounds have been active ingredients in many formulations used in traditional folk medicine as well as in increasingly popular modern natural medicine [12,13]. Therefore, they represent a promising group of chemical compounds in the search for new drugs and combinations of existing medications with natural substances, especially since rising antimicrobial resistance is one of the key challenges facing 21st-century medicine. The general structural formulas of selected groups of antibiotics (Figure 1) and the structural formulas of selected carboxylic acids (Figure 2) are shown in the figures below.

The aim of this study is to evaluate the impact of cloxacillin, as well as cinnamic acid and its derivatives, on biofilm-formation capacity. Additionally, the effect of combinations of antibiotics and carboxylic acids on inhibiting the biofilm-formation process will be investigated. The selected concentrations for this assessment were chosen based on their recognition as exhibiting a synergistic effect [14].

## 2. Results and Discussion

### 2.1. Biofilm-Forming Ability

**Figure 3:**
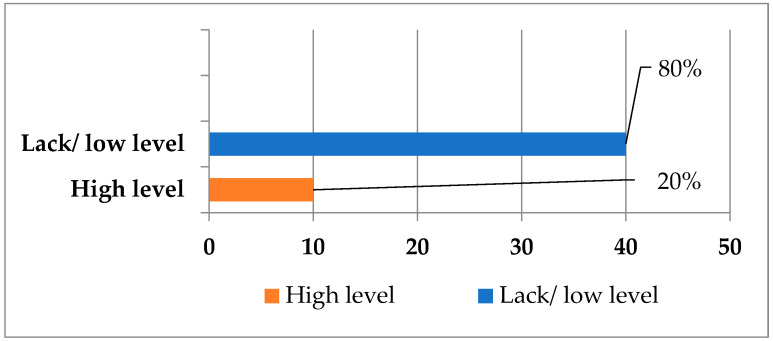
Percentage of strains with high biofilm-formation capacity.

All 50 strains of *Staphylococcus epidermidis* were subjected to a phenotypic assessment of their biofilm-formation ability. This analysis allowed for the classification of the tested sample into two populations: strains with a high capacity for biofilm formation and strains that either do not produce biofilms or have a low phenotypic capacity for biofilm formation. The results obtained are presented in the diagram below (Figure 3). Further studies were conducted exclusively on strains exhibiting a strong phenotypic ability to form biofilms.

In our study, 40 strains (80%) demonstrated no or low biofilm-formation capacity, while 10 (20%) exhibited a high capacity for biofilm production. Cabrera-Contreras et al. analyzed a group of *S. epidermidis* strains isolated from hospital infections in Mexico. This population was almost five-times larger (245 strains) than the population examined in the present study. However, the Mexican researchers obtained a similar percentage of strains with phenotypic biofilm-formation ability, amounting to 34% of the population. It is worth noting that both studies employed phenotypic methods [15]. Soumya et al. analyzed 173 clinical samples, including 90 strains identified as *S. epidermidis*. Using phenotypic methods, they classified 11% of the strains as having a strong biofilm-forming capacity [16]. A team led by Kaiser et al. investigated a modified Congo Red Agar medium used for phenotypic assessment of biofilm-formation capacity. In their study, 26.3% of the strains maintained biofilm-formation capacity after 48 h of aerobic incubation, while only 5.2% retained this ability after 48 h of microaerophilic incubation. It is important to note that all strains exhibited genotypic potential for biofilm production [17].

### 2.2. Antimicrobial and Antibiofilm Activity

**Figure 4:**
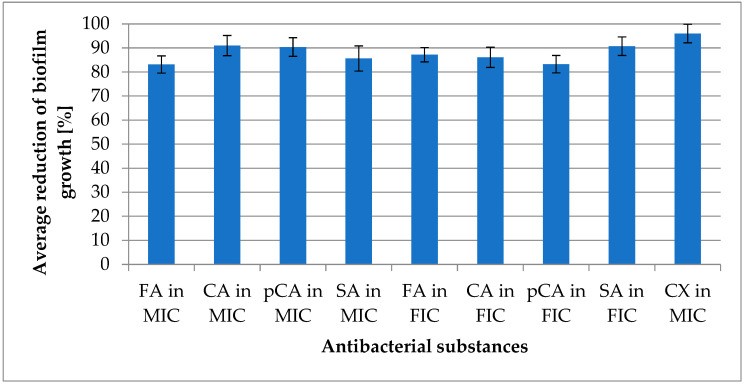
Average reduction of planktonic form growth; “in MIC”—concentration of substance in minimum inhibitory concentration (MIC) of substance; “in FIC”—concentration of substance in fractional inhibitory concentration (with cloxacillin); CA—cinnamic acid; FA—ferulic acid; pCA—p-coumaric acid; SA—sinapic acid; CX—cloxacillin.

This study compared antimicrobial activity against both planktonic forms and biofilm-associated cells. In the assessment of anti-planktonic activity conducted in Müller–Hinton medium, a reduction in microbial growth was observed, ranging from 83.15% (±3.554) for ferulic acid to 90.98% (±4.199) for cinnamic acid. For p-coumaric acid, the reduction in growth was 90.39% (±3.893), while sinapic acid achieved a reduction of 85.64% (±5.193). Comparable values were obtained for combinations of acids with the antibiotic. The reduction in growth ranged from 83.27% (±3.624) for the combination of p-coumaric acid with cloxacillin to 90.75% (±3.853) for the combination of sinapic acid with cloxacillin. The combination of cinnamic acid with cloxacillin reduced growth by 86.09% (±4.203), while the combination of ferulic acid with cloxacillin resulted in an 87.18% (±2.973) reduction. Details of the concentrations utilized in this study can be found in Table S1 [14]. The obtained results, presented as growth reduction percentages, illustrate the effectiveness of the tested acids and their combinations with the antibiotic in inhibiting microbial growth compared to control groups. The results are detailed in Figure 4.

**Figure 5:**
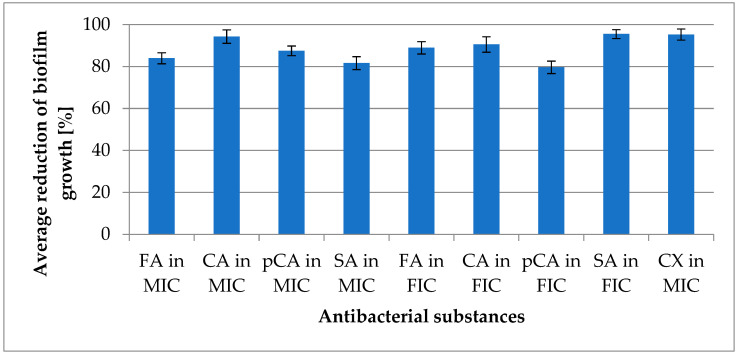
Average reduction of biofilm growth; “in MIC”—concentration of substance in minimum inhibitory concentration (MIC) of substance; “in FIC”—concentration of substance in fractional inhibitory concentration (with cloxacillin); CA—cinnamic acid; FA—ferulic acid; pCA—p-coumaric acid; SA—sinapic acid; CX—cloxacillin.

Anti-biofilm activity was determined using TSB medium supplemented with 0.25% glucose. The reduction ranged from 81.67% (±3.068) for sinapic acid to 94.30% (±3.186) for cinnamic acid. Ferulic acid reduced biofilm growth by 83.98% (±2.628), while p-coumaric acid achieved a reduction of 87.54% (±2.302). Combinations of selected acids with cloxacillin resulted in biofilm growth reductions ranging from 79.65% (±2.958) for p-coumaric acid to 95.51% (±2.135) for sinapic acid. The reduction value for ferulic acid was 88.95% (±2.958), and for cinnamic acid, it was 90.53% (±3.682). Cloxacillin alone demonstrated a reduction in planktonic forms of 96.01% (±3.852) and biofilm reduction of 95.28% (±2.628). These results are presented in Figure 5.

The activity against planktonic forms was determined in the study by Malheiro et al. [18]. They reported a significant reduction in the planktonic forms of *Staphylococcus aureus* for cinnamic acid and a relatively weak reduction for coumaric and ferulic acids. Research by Borges et al. [19] highlighted the impact of ferulic acid on both the inhibition of *Staphylococcus aureus* growth and the reduction of bacterial motility. The broad antimicrobial activity of cinnamic acid and its derivatives is frequently reported in the literature. However, studies on the anti-biofilm effects of specific carboxylic acids are less common.

Borges and colleagues examined the influence of ferulic acid on the biofilm development of several bacterial species, including *Staphylococcus aureus*. They observed no significant reduction in biofilm mass, despite a decreased metabolic activity of the biofilm. In their work on ferulic acid and its derivatives, Ergun et al. [20] observed high minimum inhibitory concentration (MIC) values for the tested strains, yet they recorded very promising anti-biofilm activity for one of the derivatives.

Yue et al. [21] analyzed a complex consisting of conjugated hydroxypropyl chitosan derivatives with cinnamic acid. Using advanced techniques such as confocal laser scanning microscopy, they observed a significant impact on the viability of bacterial cells within the biofilm structure. Albano et al. [22] conducted studies on a compound closely related to cinnamic acid, namely cinnamaldehyde. They reported lower MIC values against planktonic forms (300–500 µg/mL) and a lower reduction in biofilm growth (with a reduction of 73% at 70% of MIC). In this study, reduction values were obtained at half the MIC concentration.

Cinnamic acid and its derivative acids are compounds frequently described as having antimicrobial properties. However, it should be noted that the minimum inhibitory concentration (MIC) and fractional inhibitory concentration (FIC) values are high/very high, ranging from 256 to 4096 µg/mL. Toxicological data obtained from substance safety data sheets indicate an LD50 of 2500 mg/kg body weight for cinnamic acid and 2850 mg/kg body weight for p-coumaric acid in rats upon oral administration [23,24].

Oxacillin, a semisynthetic penicillin with a narrow spectrum of activity and resistance to staphylococcal penicillinases, is structurally similar to cloxacillin. De Oliveira et al. [25] investigated the anti-biofilm activity of beta-lactam antibiotics. They determined the minimum bactericidal concentration (MBC) for biofilm cells of oxacillin. For *Staphylococcus aureus* strains, MBC values ranged from 8 to >256 µg/mL. For *Staphylococcus epidermidis*, the value was 256 µg/mL or higher, while for other coagulase-negative staphylococci, it ranged from 8 to >256 µg/mL. The authors also referenced high values reported in other publications, which seem to support the findings in this study.

The activity of antibiotics in combination with natural compounds (including all the acids discussed in this publication) was examined by Kincses et al. [26]. They investigated the impact of antibiotics from the fluoroquinolone, tetracycline, aminoglycoside, and aminopenicillin classes, in combination with various compounds. They demonstrated a two- to four-fold decrease in MIC values for selected antibiotic combinations, highlighting the significant therapeutic potential of such combinations. Unfortunately, the antibacterial activity of combinations of selected acids with beta-lactam antibiotics and their anti-biofilm activity was not assessed in their study.

### 2.3. Confocal Imaging of Staphylococcus epidermidis Biofilm

Confocal microscopy is used for visualizing bacterial biofilm [27]. By staining bacterial cells with appropriate fluorescent dyes, it is possible to distinguish between live and dead cells. Among the 50 tested strains of *S. epidermidis*, a representative strain with a strong ability to produce biofilm was selected. The effects of various concentrations of cinnamic acid and its derivatives on biofilm production capacity and the number of dead bacterial cells on the surface of the vessel were investigated.

**Figure 6:**
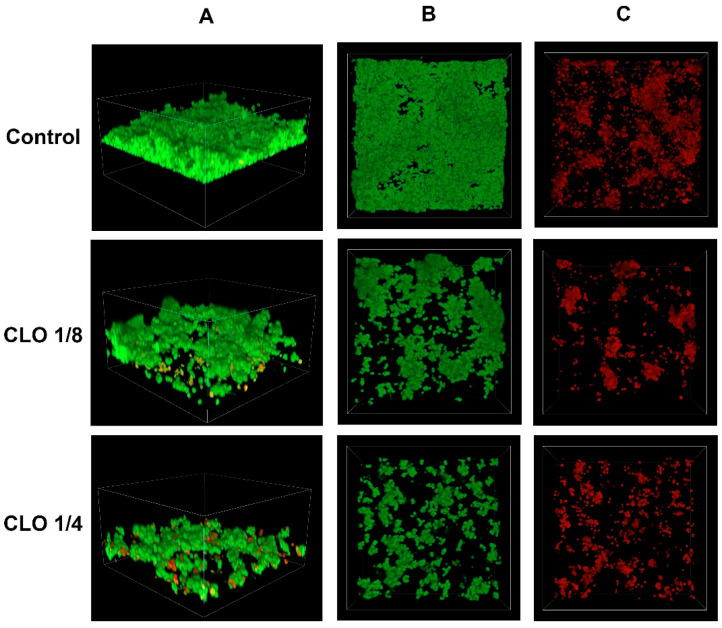
Confocal images of untreated (control) *Staphylococcus epidermidis* biofilm and the biofilm exposed to cloxacilline (CLO) at various dilutions: CLO 1/8—cloxacilline at 1/8 MIC, CLO 1/4—cloxacilline at 1/4 MIC; (**A**) z-stack 3D reconstruction; (**B**) top view in SYTO 9 channel (green); (**C**) top view in propidium iodide channel (red).

The biofilm inhibition potential of cinnamic acid and its derivatives was compared to the activity of the antibiotic cloxacillin. The figure below illustrates the effect of cloxacillin at sub-MIC concentrations on the growth of *S. epidermidis* (Figure 6).

**Figure 7:**
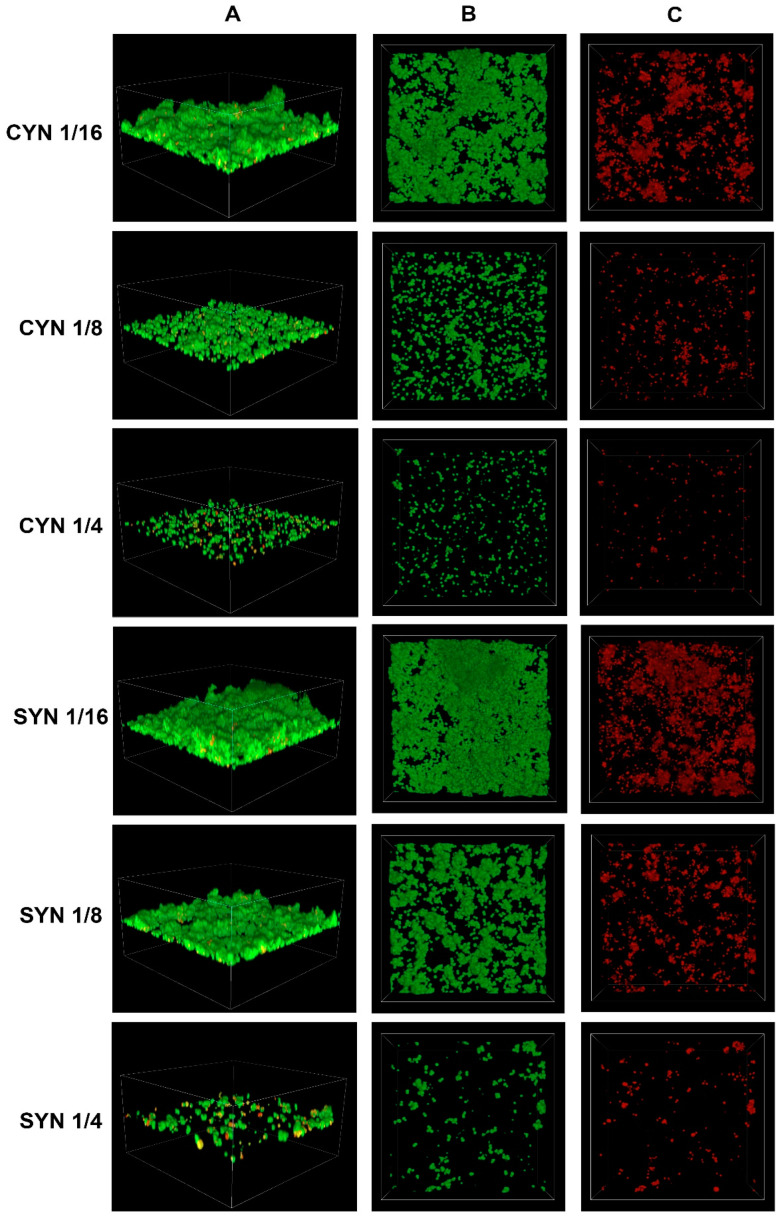
Confocal images of *Staphylococcus epidermidis* biofilm exposed to cinnamic acid (CYN) or sinapic acid (SYN) at various dilutions: e.g., CYN 1/4—cinnamic acid at 1/4 MIC; (**A**) z-stack 3D reconstruction; (**B**) top view in SYTO 9 channel (green); (**C**) top view in propidium iodide channel (red).

**Figure 8:**
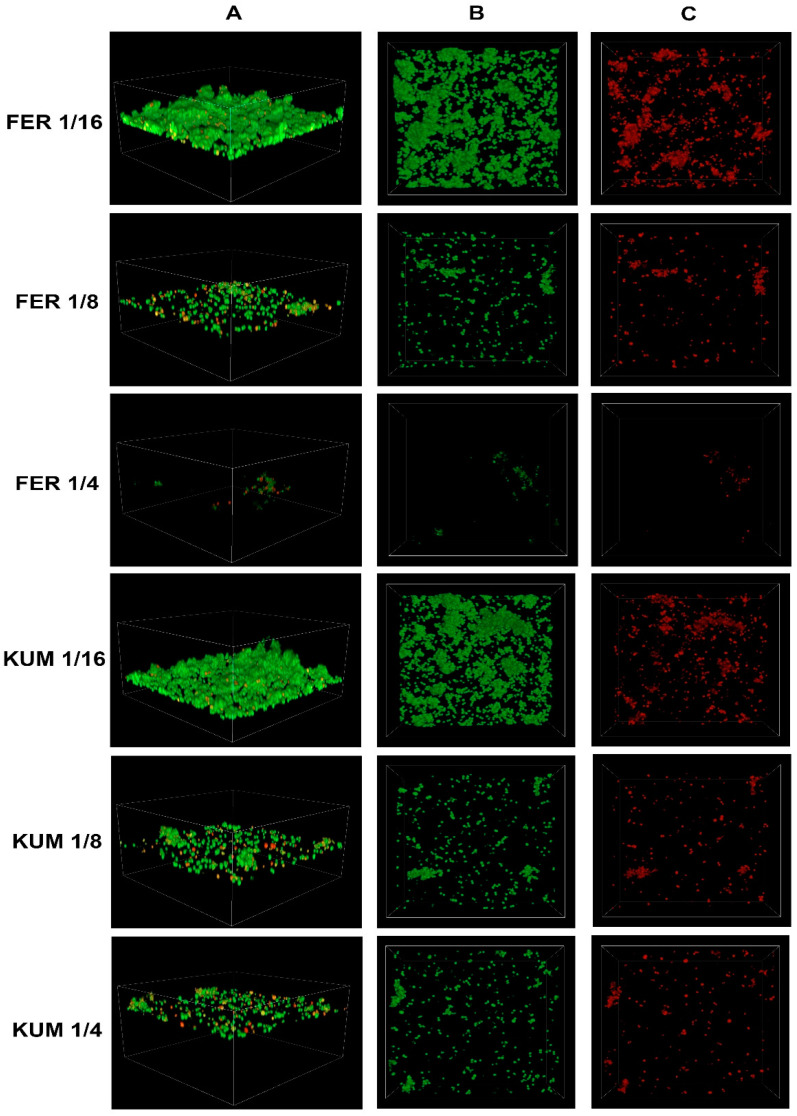
Confocal images of *Staphylococcus epidermidis* biofilm exposed to ferulic acid (FER) or p-coumaric acid (KUM) at various dilutions: e.g., FER 1/4—ferulic acid at 1/4 MIC; (**A**) z-stack 3D reconstruction; (**B**) top view in SYTO 9 channel (green); (**C**) top view in propidium iodide channel (red).

The figure below shows the effect of cinnamic acid and its derivatives at individual concentrations on the growth of *S. epidermidis* (Figure 7 and Figure 8). There is a visible effect reducing the number of bacterial cells, directly proportional to the concentration of the substance used.

In all cases, a reduction in the number of cells is observed with increasing concentrations of the respective acids. To better illustrate the relationship between the decrease in the number of viable cells and the increase in the number of dead cells as the concentrations of the tested substances increase, the values were expressed as sub-MIC concentrations (e.g., 1/4 KUM, 1/8 FER).

Confocal microscopy has been applied in studies focusing on the effects of derivatives of cinnamic acid and other compounds. In both cases, a similar impact on the reduction of the formed biofilm was achieved [21,28]. Future research stages will focus on characterizing the antibiofilm efficacy of combinations comprising derivatives and antibiotics.

## 3. Materials and Methods

### 3.1. Bacterial Cultures

Bacterial cultures were obtained from patients with vascular catheter infections in the Silesian Voivodeship. The species identity of all microorganisms (50 strains) was confirmed using MALDI-TOF mass spectrometry—Vitec MS Prime^®^ system (bioMérieux, Craponne, France). The identification was conducted using the VITEK MS Prime^®^ system (bioMérieux, Craponne, France) according to the manufacturer’s instructions. All microorganisms that qualified for the study were identified as *Staphylococcus epidermidis*.

### 3.2. Biofilm-Formation Assay

Biofilm formation was assessed using 96-well microtiter plates (Thermo Electron Corp., Vantaa, Finland). Each well received 150 µL of TSB medium with 5% glucose and 50 µL of bacterial suspension with a turbidity of 0.5 on the McFarland scale (1–2 × 10^8^ CFU/mL) in a 0.9% NaCl solution. Three wells were designated for each strain.

**Figure 9:**
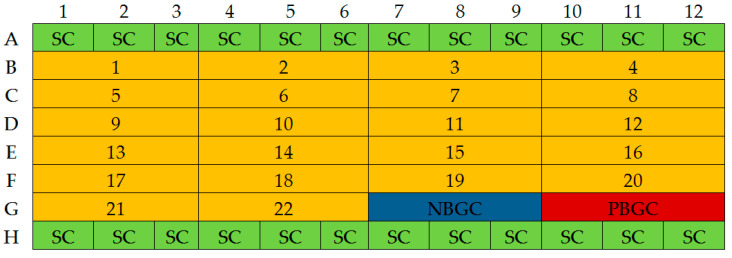
Layout of the assay plate; SC—sterility control; NBGC—non-biofilm-producing strain growth control; PBGC—biofilm-producing strain growth control; numbers: sequential numbers of the tested strains.

For sterility control, a solution of physiological saline was added to the TSB medium with 0.25% glucose. The cultures were incubated at 37 ± 1 °C for 18 h. The layout of the assay plate is illustrated in the figure below (Figure 9).

After incubation, the cultures were subjected to staining using crystal violet according to the modified Christensen method [29]. For this purpose, the growth medium was removed, and the wells were washed three times with phosphate-buffered saline (PBS, pH = 7.2) to remove remaining planktonic forms from the culture. After drying (30 min at 37 °C), 200 µL of 1% crystal violet solution (MERCK/Sigma-Aldrich, Darmstadt, Germany) was added to each well. Staining was performed for 10 min at 25 °C. After staining, the wells were washed four times with deionized water and then dried for 30 min at 37 °C. Next, 100 µL of 95% isopropanol in 1 M HCl was added, and the optical density was read at a wavelength of λ = 570 nm using a Multiskan EX (Thermo Electron Corp., Vantaa, Finland) microplate reader.

The obtained results were divided into two populations: those with high biofilm-formation capacity and those with a lack of or low biofilm-formation capacity.

### 3.3. Assessment of Anti-Biofilm Activity of Combinations

A 96-well microtiter plate was used for the assessment. Each well was filled with either 180 µL of TSB medium with 0.25% glucose containing the antibiotic/phenolic acid or a mixture of 90 µL of TSB medium with 0.25% glucose containing the antibiotic at a concentration twice the MIC and 90 µL of TSB medium with 0.25% glucose containing the selected acid at a concentration twice the MIC. The MIC values for the antibiotics and acids, as well as the FIC values for the combinations, were determined in a previous stage of the study [14].

Then, 20 µL of bacterial suspension with a turbidity of 0.5 on the McFarland scale (1–2 × 10^8^ CFU/mL) in 0.9% NaCl solution was added to each well. Three wells were designated for each strain.

**Figure 10:**
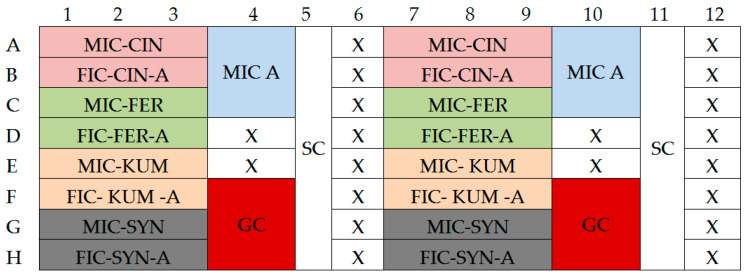
Layout of the assessment of the chemical compounds’ impact on biofilm reduction; MIC-X—growth with the addition of acid/antibiotic at MIC concentration; FIC X-A—growth with the addition of acid and antibiotic at FIC concentration; GC—growth control; SC—sterility control; CIN—cinnamic acid; FER—ferulic acid; KUM—p-coumaric acid; SYN—sinapic acid; A—antibiotic.

For sterility control, 20 µL of physiological saline was added to 180 µL of TSB medium with 0.25% glucose. The growth control was prepared by adding 20 µL of bacterial suspension with a turbidity of 0.5 on the McFarland scale (1–2 × 10^8^ CFU/mL) to 180 µL of TSB medium with 0.25% glucose. The cultures were incubated at 37 ± 1 °C for 18 h. The layout of the assay plate is shown in the figure below (Figure 10).

After an 18-h incubation, the cultures were stained using the modified Christensen method. The procedure was identical to that performed in the earlier studies. Subsequently, the biofilm growth was quantified as a percentage of the growth control, and the percentage reduction in biofilm mass was calculated.

### 3.4. Visualization of Bacterial Biofilm

A Nikon Eclipse Ti-E A1R-Si confocal laser scanning microscope (Nikon Instruments, Amsterdam, The Netherlands) and Nikon NIS Elements AR 4.51 software were used for the 3D visualization of biofilm architecture and examination of biofilm viability. The samples on coverslips were fixed with 4% paraformaldehyde and then stained with Syto^TM^ 9 green, fluorescent nucleic acid stain (Invitrogen, Eugene, OR, USA) and bacteria stain propidium iodide (MERCK/Sigma-Aldrich, Darmstadt, Germany), according to the manufacturer protocol. SYTO 9 dye (visualized in green channel) penetrates cells with both intact and compromised membranes, while propidium iodide (visualized in the red channel) only stains cells with damaged membranes. The samples were imaged with an 60× oil immersion objective using the Z-stack function.

## 4. Conclusions

1. In the studied population of *Staphylococcus epidermidis*, 20% of the strains exhibited a high ability to form biofilm.
2. The highest activity against planktonic forms was observed for cloxacillin, cinnamic acid, and p-coumaric acid.
3. The most significant anti-biofilm activity was demonstrated by cloxacillin, cinnamic acid, and a combination of sinapic acid and cloxacillin, as assessed by the FIC (fractional inhibitory concentration) index.
4. All tested substances exhibited strong activity against planktonic forms, and nearly all displayed the ability to reduce biofilm formation by over 80% compared to the growth control. The exception was the combination of p-coumaric acid and cloxacillin at the FIC value, which showed a reduction capacity of 79.65%.
5. The integration of antibiotics with selected derivatives achieved up to a 16-fold decrease in growth-inhibitory concentrations. It is therefore recommended to consider further work on the combinations of cinnamic acid derivatives and antibiotics, which may potentially lead to the development of new combinations with therapeutic potential.
6. Confocal laser scanning microscopy revealed an increase in the percentage of dead cells with the increasing concentration of cinnamic acid and its derivatives.

## Data Availability

Data are contained within the article.

## Supplementary Materials

The following supporting information can be downloaded at: https://www.mdpi.com/article/10.3390/molecules30030660/s1, Table S1: MIC and FIC values for *Staphylococcus epidermidis* strains with the ability to produce biofilm.
