# Supplementary material for: Evaluation of the Antibacterial Activity of Cinnamic Acid and Its Derivatives: Synergistic Effects with Cloxacillin
Source: Molecules. 2025 Feb 2;30(3):660. doi: 10.3390/molecules30030660 (PMC11820451; doi:10.3390/molecules30030660)
Supplement: Supplementary file 1 [file molecules-30-00660-s001.zip › molecules-3391736-supplementary.pdf]

Strain	1			
MIC Clox	256		C Acid [μg/mL]	C Clox [μg/mL]
MIC CIN	4096	FIC CIN	256	16
MIC FER	4096	FIC FER	256	32
MIC SIN	4096	FIC SIN	512	16
MIC pCU	4096	FIC pCU	512	16
Strain	2			
MIC Clox	256			
MIC CIN	4096	FIC CIN	256	2
MIC FER	4096	FIC FER	512	2
MIC SIN	4096	FIC SIN	512	2
MIC pCU	4096	FIC pCU	256	2
Strain	3			
MIC Clox	128			
MIC CIN	2048	FIC CIN	256	4
MIC FER	4096	FIC FER	256	4
MIC SIN	4096	FIC SIN	512	4
MIC pCU	4096	FIC pCU	256	8
Strain	4			
MIC Clox	256			
MIC CIN	4096	FIC CIN	512	16
MIC FER	4096	FIC FER	256	8
MIC SIN	4096	FIC SIN	512	8
MIC pCU	4096	FIC pCU	512	8
Strain	5			
MIC Clox	128			
MIC CIN	4096	FIC CIN	512	16
MIC FER	4096	FIC FER	512	16
MIC SIN	4096	FIC SIN	512	16
MIC pCU	4096	FIC pCU	512	16
Strain	6			
MIC Clox	1			
MIC CIN	4096	FIC CIN	256	0.5
MIC FER	4096	FIC FER	512	0.5
MIC SIN	4096	FIC SIN	512	0.5
MIC pCU	4096	FIC pCU	512	0.5
Strain	7			
MIC Clox	1			
MIC CIN	4096	FIC CIN	512	0.5
MIC FER	4096	FIC FER	512	0.5
MIC SIN	4096	FIC SIN	512	0.5
MIC pCU	4096	FIC pCU	512	0.5
Strain	8			
MIC Clox	256			
MIC CIN	4096	FIC CIN	256	32
MIC FER	4096	FIC FER	256	16
MIC SIN	4096	FIC SIN	512	32
MIC pCU	4096	FIC pCU	512	16

Strain	9			
MIC Clox	256			
MIC CIN	4096	FIC CIN	512	64
MIC FER	4096	FIC FER	1024	64
MIC SIN	4096	FIC SIN	512	32
MIC pCU	4096	FIC pCU	512	16
Strain	10			
MIC Clox	1			
MIC CIN	4096	FIC CIN	256	0.5
MIC FER	4096	FIC FER	256	0.5
MIC SIN	4096	FIC SIN	256	0.5
MIC pCU	4096	FIC pCU	256	0.5

Table S1. MIC and FIC values for *Staphylococcus epidermidis* strains with the ability to produce biofilm; MIC - Minimal Inhibitory Concentration of substance [μg/mL]; FIC - Fractional Inhibitory Concentration of substances - in combination [μg/mL]; C Clox - Concentration of cloxacillin in FIC; C Acid - Concentration of acid in FIC; Clox - cloxacillin; CIN - cinnamic acid; FER - ferulic acid; SIN -sinapic acid; pCU - p-coumaric acid [14].
